# miRNA as biomarker in lung cancer

**DOI:** 10.1007/s11033-023-08695-9

**Published:** 2023-09-23

**Authors:** Esperanza Salcedo Lobera, Macarena Arroyo Varela, Rafael Larrosa Jimenez, Rocio Bautista Moreno

**Affiliations:** 1 U.G.C. Medico-Quirurgica de Enfermedades Respiratorias, Hospital Regional Universitario de Malaga, Malaga, Spain; 2 Department of Computer Architecture, University of Malaga, Malaga, Spain; 3 Andalusian Platform for Bioinformatics at SCBI, University of Malaga, Malaga, Spain

**Keywords:** MicroRNA, High-throughput sequencing, RNA-seq, Biomarker, Lung cancer

## Abstract

Lung cancer has a high prevalence and mortality due to its late diagnosis and limited treatment, so it is essential to find biomarkers that allow a faster diagnosis and improve the survival of these patients. In this sense, biomarkers based on miRNAs have supposed a considerable advance. miRNAs, which are small RNA sequences, can regulate gene expression, so they play an essential role not only as a diagnostic biomarker but also as a therapeutic and prognostic one. Also, miRNA biomarkers can be obtained from liquid biopsies, which are less intrusive than lung biopsies, and have better accessibility, safety and repeatability, which allows using those biomarkers both for diagnosis and monitoring of patients. In this review, we highlight the importance of miRNAs and collect the existing evidence of their relationship with lung cancer.

## Introduction

Lung cancer (LC) or bronchogenic cancer is a disease with a high prevalence and mortality. According to the World Health Organization (www.who.int), lung cancer is the most commonly diagnosed cancer in men and the second most frequently diagnosed cancer in women [1]. Although its knowledge has been highly developed over the last few years, the WHO estimates that LC is still responsible for approximately 18% of all new cancer cases and 22% of all cancer-related deaths worldwide, with a survival rate of around 20.5%. Regarding his histological classification, most lung neoplasms are of epithelial origin. Approximately 85% of LC are classified as non-small cell lung cancer (NSCLC), 15% as small cell lung carcinoma (SCLC) and 5% as large cell carcinoma (LCG). Inside NSCLC, the most frequent histological groups are adenocarcinoma (ADC) and squamous cell carcinoma (SCC). On the other hand, this classification of lung cancer is continuously updated, chiefly thanks to the knowledge acquired about its molecular development; hence having an accurate classification is vital to perform a correct diagnosis that allows subsequent specific treatment. In this sense, using biomarkers is becoming a primary classification method since it enables the characteristics of each type of lung cancer to be defined. Regarding his clinical incidence, the incidence of each of these histological groups has been changing over the years. Previously, SCC was the most frequent due to its relationship with the smoking habit; however, cases of ADC have now increased to the point of being the first in frequency and generally without a causal relationship with tobacco. Each one of the previous histological groups presents a series of peculiarities. ADC, characterized as a neoplasm by glandular differentiation, is the most prevalent, being more frequent in women and non-smokers. ADC is located at the peripheral level and has a greater tendency to distant metastasis. SCC, also known as carcinoma epidermoid, is characterized by forming intercellular bridges and keratinization. It is directly related to tobacco, being its most important causal agent. It is centrally located and sometimes presents as central necrosis. The processes of metastasis are common in this type of tumour. SCLC can be diagnosed due to a neuroendocrine differentiation, being neuroendocrine neoplasia the most frequent pulmonary disease related to the smoking habit. It has a high-speed evolution, is localized centrally, and is associated with paraneoplastic syndromes. It is usually diagnosed in advanced stages, which makes eradication therapy impossible. Finally, LCG is an undifferentiated epithelial carcinoma that does not meet the criteria of any of the above types, so the diagnosis is made by exclusion. It appears as a mass periphery in smoking patients [2].

All these neoplasms could be made into preinvasive lesions, such as hyperplasia, metaplasia, dysplasia and carcinoma in situ, and they can evolve into lung carcinoma. This evolution has a molecular basis formed by numerous genetic and epigenetic changes within the cell nucleus, causing a high susceptibility to undergo mutations that will entail a series of pathophysiological alterations, mainly unlimited cell proliferation, resistance to growth inhibitory signals, evasion of apoptosis, angiogenesis and tumour invasion [3].

The diagnosis of LC is usually conducted on lung tissue, with the consequent invasion of the organ. However, despite the improvements in early diagnosis of lung cancer, most lung cancers are diagnosed at an advanced stage. Hence in recent years, much effort has been invested in finding biomarkers on liquid biopsies that would allow an early diagnosis without the need for aggressive interventions. Therefore, the identification of novel diagnostic biomarkers or treatment strategies is critical and essential for the control of lung cancer [4].

## Biomarkers in cancer

Given the large heterogeneity in clinical across cancer patients, personalised treatments are difficult to introduce. Many of the targeted treatments against any LC act on key therapeutic points identified by genome mutations; however, still benefit a low number of patients. Our understanding of cancer at the molecular level has resulted in a shift from characterising tumours. Hence, the use of differentiating molecules, such as biomarkers, could improve detection in the early stage of LC. A biomarker is defined as an objectively measurable and evaluable biological characteristic that serves as an indicator of biological processes. So, the ideal biomarkers for cancer should be helpful for diagnosis, prognosis, and/or response to treatment and, therefore, should have high sensitivity, specificity, and positive predictive value [1].

**Fig. 1:**
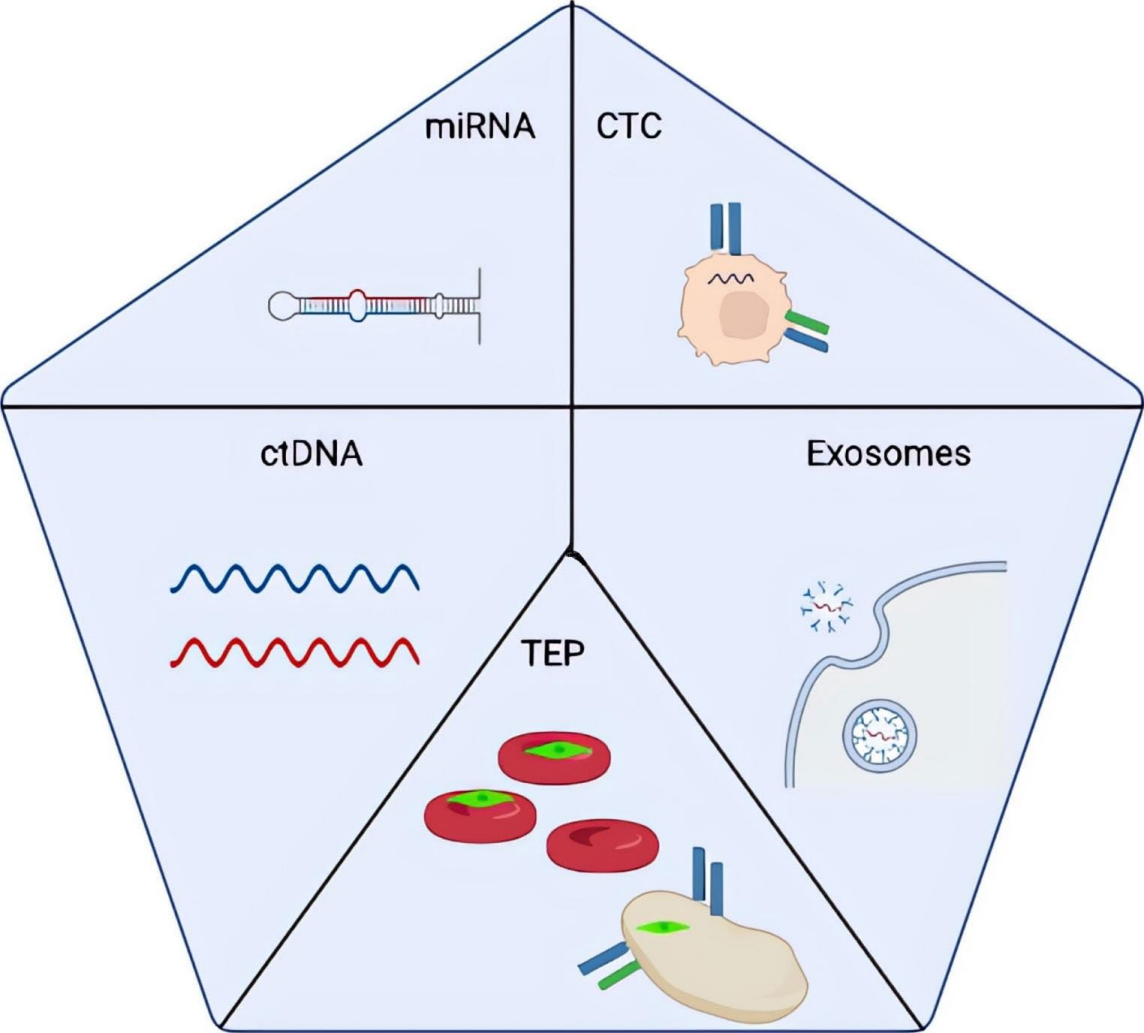
Molecules that can be used as biomarkers. miRNA: small RNA from tumour cells, CTC: circulating tumour cells, CtDNA: DNA residues from tumour cells, TEP: platelets containing tumour RNA, Exosomes: extracellular vesicles secreted by tumourcells

Thanks to the different advances in the knowledge of the molecular biology of cancer and its genetics, it has been possible to determine various types of molecules, in addition to genome mutations, that can be used as biomarkers in cancer [2], as seen in Fig. 1. Among these molecules, they are worth highlighting (I) circulating tumour cells (CTC), cells derived from the tumour that is free in the bloodstream so that they can be detected in liquid biopsies. (II) Circulating tumour DNA (CtDNA), DNA residues released by the tumour tissue after processes such as necrosis, apoptosis, or the secretion of vesicles, can be detected in the blood. (III) The platelets formed by tumours (TEP), that are platelets that contain tumour RNA, which could regulate tumour growth, invasion and distant metastasis. (IV) Exosomes, extracellular vesicles 40-100 nm in size, are secreted by tumour cells into the bloodstream. Many studies indicate the importance of these exosomes’ vesicles for communication between the different neoplastic cells, transferring information through various mechanisms, such as ligand-receptor interaction. (V) miRNA are small molecules of non-coding RNA transcribed after changes in the regulation of neoplastic cells, excreted into the bloodstream where they are free and are, therefore, easily detectable.

## Liquid biopsy

The tumour tissue sample through surgical biopsy is the technique more used, although we can not forget it is invasive. Surgical biopsies present a series of drawbacks such as are: (1) the inaccessibility of the tumour on some occasions due to its location; (2) the difficulty in monitoring the patients undergoing active cancer treatment; (3) the complications related to the procedure, such as infections and haemorrhages; and finally, (4) high cost of the technique [3, 4].

For this reason, over the last few years, studies have emerged about a new way of obtaining samples without invading the tissues, liquid biopsies, that could improve the diagnostic or prognosis of a disease with high mortality as LC. In this sense, personalized medicine is gaining interest in using these liquid biopsies, as it could provide an early diagnosis, making possible more accessible cancer treatment. Even so, surgical biopsies are still used customarily, as they allow taking a direct sample of the tumour, so it is the standard technique for diagnosing neoplasms. It is mainly used to search for specific mutations that could provide targeted therapies.

On the other hand, other procedures are used due to the complexity and risk of performing tissue biopsies. Among those diagnostic tests, the main one is computer tomography (CT), which has a drawback in the extensive use of radiation. Another option is magnetic resonance imaging (MRI), which, despite not having radiation, is inefficient in monitoring the disease due to the low quality of information that it provides.

For all of the above, liquid biopsies are on the rise for the detection of tumour formation, thanks to their multiple advantages, among which is the ease of acquiring samples being a non-invasive and repeatable technique. All the interest in this type of biopsy lies in the analysis of different biomarkers, such as the circulating tumour cells (CTCs), the circulating tumour DNA (ctDNA) or miRNAs that are It is found in exosomes from blood, plasma, serum or sputum.

However, these biomarkers also have some disadvantages. CtDNA has a short half-life and is presented at low concentration and low sensitivity; the CTC is found in low concentration and low specificity in the early stages of the disease; the miRNA is included in exosomes, and they need standardization although they have a long half-life [5]. Despite the previous, there are numerous studies on the usefulness of liquid biopsies since its combination with chest CT could detect phase tumours earlier, either in the diagnostic phase or a follow-up [6]. Using liquid biopsies would also be helpful to carry out studies in patients with advanced disease or, as we have indicated previously, with difficulty in taking a tissue biopsy, thus avoiding the lack of treatment [7].

## Role of miRNA in lung cancer

**Fig. 2:**
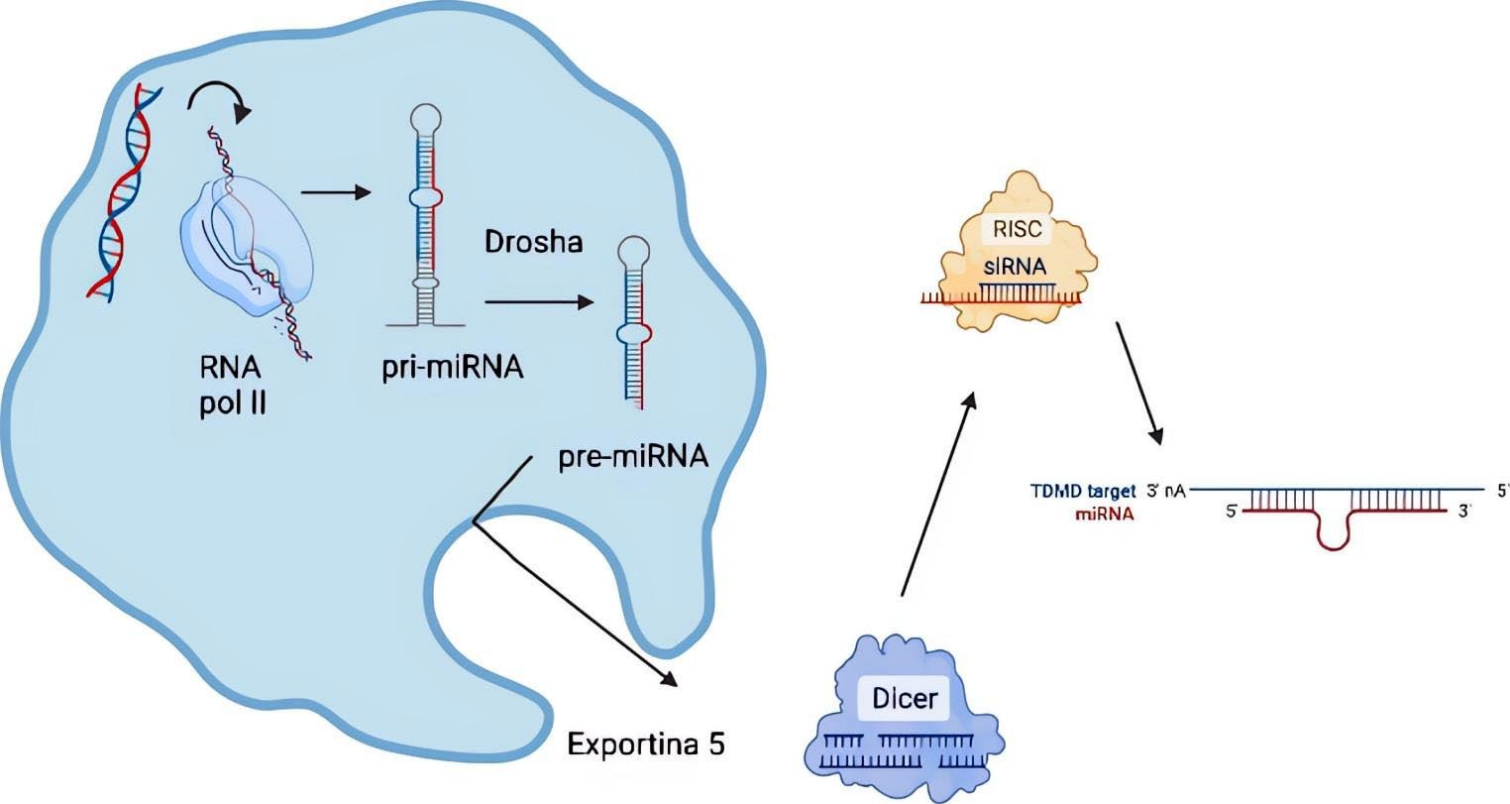
miRNA biogenesis and mechanism of action. The process begins with transcription in the cell nucleus, the Drosha enzyme cleaves the pri-miRNA to produce the precursor-miRNA (pre-miRNA), which is transported thanks to exportin 5 to the cytoplasm, where it binds with the cell nucleus. DICER protein to complete its maturation and forms a complex with the RISC protein whose objective is to inhibit gene translation

To understand the importance of miRNAs, it is necessary to know their role as regulators of post-transcriptional gene expression. The miRNAs were discovered in 1993 as negative regulators of the messenger RNAs (mRNA). These miRNAs are characterized by their relative stability, diminutive size, and great control over the regulation of gene expression. Those have multiple functions at the cellular process regulation level, including cell growth and differentiation, apoptosis and tumour progression, among others [8]. Thanks to those, there are multiple lines of research where their importance is being observed, as not only diagnostic targets for cancer but also therapeutic and forecasts. miRNAs biogenesis is widely studied. The process begins with the transcription of the miRNA in the cell nucleus, which later is transported to the cytoplasm where it splits into two mature strands; one binds to the mRNA to perform its function as a negative regulator of the gene expression while the other strand is degraded [9] (Fig. 2). A summary of the most critical miRNA in lung cancer is shown in Table 1.

## miRNA as oncogenes and tumour suppressors

The let-7 family, one of the most studied, plays an essential role in tumour progression since it causes a decrease in the expression of DICER, an RNase III family protein. This downregulate is related to a worse prognosis in non-small cell carcinomas (ADC and SCC). However, it is not the only miRNA; there are others, such as the hsa-miR-34 family, in which low expression causes an increase in the expression of different genes with increased cell proliferation. In this sense, it is well known the role of the hsa-miR-21, which has been extensively studied since its overexpression inhibits the expression of some genes, causing an increase in cell multiplication, migration and a decrease in apoptosis. Others as hsa-miR-17 and hsa-miR-135 act similarly; their overexpression in small cell carcinomas (SCLC) causes an increase in cell proliferation. The overexpression of hsa-miR-25 correlates with an increase in cell proliferation and invasion. Others, such as hsa-miR-126, hsa-miR-100, hsa-miR-145, present a higher expression level in lung cancer, having an inverse relationship with *VEGF* family *(VEGF-A, VEGF-B, VEGF-C, VEGF-D and PlGF)* [9]. We remember that they play a vital role in the processes of blood vessel formation in embryonic development and in pathological angiogenesis and lymphangiogenesis, allowing the tumour to grow exponentially. Others, such as hsa-miR-200, hsa-miR-375 and hsa-miR-486 are related to the development of lung adenocarcinoma [10].

## miRNAs as diagnostic biomarker

One of the biggest problems in the classification of lung cancer is its later diagnosis, which drastically reduces the current therapeutic options. Some studies focus on how liquid biopsies could become a novel tool for early lung cancer diagnosis. These studies show us how the sensitivity and specificity of the miRNA expression could be used as biomarkers.

In this sense, different families of miRNAs have been studied as diagnostic biomarkers. So, the overexpression hsa-miR-21, hsa-miR-200, hsa-miR-210, hsa-miR-182 and miR-183 are related to tumour progression, while the repression of the hsa-miR-30 or hsa-miR-451 has the same behaviour [11].

In the case of hsa-miR-21, its expression in sputum has a sensitivity of 70% and a specificity of 100% in the detection of lung cancer, compared to others that have been studied in plasma, such as hsa-miR-210 or miR-126, which a sensitivity of 86% and specificity of 97%, or in serum where the miR-200b presents a high precision to differentiate between patients with lung cancer versus those without [12, 13].

On the other hand, several miRNAs allow differentiation between the types of lung neoplasms. For example, hsa-miR-205 can distinguish between squamous cell carcinoma (SCC) and non-squamous cell carcinoma (NSCC). Other researchers have developed genetic signatures formed by let-7, hsa-miR-25, hsa-miR-34, and hsa-miR-191, differentiating SCC from AD. It even has been described how hsa-miR- 378, hsa-miR-379, hsa-miR-30, or hsa-miR-200 could differentiate between AD and pulmonary granulomas [9].

**Table 1:** Significant regulated miRNAs in lung cancer tumour tissue and body fluids of patients. D/T/P means diagnostic, treatment and prognostic. In the sample column P, T, S, Se means Plasma, Tissue, Sputum,Serum

Biomarker	Expression	Sample	D/T/P	Ref
hsa-miR,23-b	Downregulated	T	-/-/+	[12, 13]
hsa-miR-27a-5p	Downregulated	T	+/-/-	[13]
hsa-miR-30a-3p	Downregulated	T	+/-/-	[13]
hsa-miR-30a-5p	Downregulated	T	+/-/-	[13]
hsa-miR-30c-2-3p	Downregulated	T	+/-/-	[13]
hsa-miR-30d-5p	Downregulated	T	+/-/-	[13]
hsa-miR-122a	Downregulated	T	-/-/+	[12]
hsa-miR-126	Downregulated	T, S	+/-/+	[15]
hsa-miR-145	Downregulated	S, T	+/-/-	[10]
hsa-miR-184	Downregulated	T	-/-/+	[12]
hsa-miR-206	Downregulated	T	-/-/+	[12]
hsa-miR-221	Downregulated	Se, P	+/-/+	[12, 14]
hsa-miR-299-3p	Downregulated	T	-/-/+	[12]
hsa-miR-370	Downregulated	T	-/-/+	[12, 13]
hsa-miR-372	Downregulated	S	+/-/-	[10]
hsa-miR-432	Downregulated	T	-/-/+	[12, 13]
hsa-miR-451	Downregulated	Se, P	+/+/-	[15]
hsa-miR-494	Downregulated	T	-/-/+	[12, 13]
hsa-miR-513	Downregulated	T	-/-/+	[12]
let-7a	Downregulated	Se, P	+/-/+	[9, 12, 14]
hsa-miR-15a	Upregulated	T	-/-/+	[12]
hsa-miR-17	Upregulated	P	+/-/-	[17]
hsa-miR-21	Upregulated	P, T, S, Se	+/-/+	[10, 11]
hsa-miR-24	Upregulated	P	+/-/-	[17]
hsa-miR-25	Upregulated	Se, P	+/-/-	[15]
hsa-miR-31	Upregulated	T	-/+/-	[12, 13]
hsa-miR-93	Upregulated	T	+/-/+	[14]
hsa-miR-98	Upregulated	T	+/-/-	[10, 14]
hsa-miR-100	Upregulated	T	+/-/+	[14]
hsa-miR-106a	Upregulated	P	+/-/-	[17]
hsa-miR-10b	Upregulated	T	-/-/+	[12]
hsa-miR-1254	Upregulated	Se	+/-/-	[10]
hsa-miR-125b	Upregulated	P	+/-/-	[17]
hsa-miR-128	Upregulated	P	+/-/-	[17, 18]
hsa-miR-135b	Upregulated	T	-/+/-	[12, 13]
hsa-miR-146b	Upregulated	T	-/+/+	[14]
hsa-miR-155	Upregulated	P, T, Se	+/+/+	[9, 14, 18]
hsa-miR-182	Upregulated	P, T, S	+/-/+	[15]
hsa-miR-183	Upregulated	P	+/-/-	[17]
hsa-miR-191	Upregulated	T, P	+/-/+	[14]
hsa-miR-192	Upregulated	Se, P, T	+/-/-	[15]
hsa-miR-197	Upregulated	T	+/+/-	[9, 16]
hsa-miR-199	Upregulated	P	+/-/-	[17]
hsa-miR-200b	Upregulated	S, T	-/+/-	[12, 13, 16]
hsa-miR-202	Upregulated	P	-/-/+	[12]
hsa-miR-203	Upregulated	P	+/-/-	[17]
hsa-miR-205	Upregulated	T, P	+/-/-	[10]
hsa-miR-210	Upregulated	S	+/+/-	[10]
hsa-miR-211	Upregulated	P	+/-/-	[17]
hsa-miR-326	Upregulated	T	-/+/-	[11–13]
hsa-miR-375	Upregulated	S, T	+/-/-	[10]
hsa-miR-378	Upregulated	Se	+/-/-	[9, 10]
hsa-miR-379	Upregulated	T	+/-/+	[9, 14]
hsa-miR-3917	Upregulated	T	+/-/-	[10, 14]
hsa-miR-453	Upregulated	T	-/-/+	[12]
hsa-miR-486	Upregulated	P, T, S	+/+/-	[10]
hsa-miR-511	Upregulated	T	-/-/+	[12]
hsa-miR-574-5p	Upregulated	Se	+/-/-	[10]

### miRNA as prognostic biomarker

Regarding lung cancer, not only is early diagnosis important, but it is also essential to know the prognosis of this type of neoplasm. Thanking to some studies, it has been possible observe how the overexpression or underexpression of different miRNAs are related to lower or increased survival. Firstly, the literature describes how Let-7, hsa-miR-23b, hsa-miR-199, hsa-miR-221 and hsa-miR-155 are linked to worse survival in patients with lung cancer [9]. On the other hand, hsa-miR-146b can predict overall survival in patients with SCC, while elevated expression of both miR-146b and miR-155 is associated with poor survival in this neoplasm [18]. The levels of hsa-miR-21 and hsa-miR-34 in plasma correlate with recurrence in NSCLC, and hsa-miR-10b is associated with better survival in patients with NSCLC, existing even miRNA that after more than seven days of tumour resection continues to appear as hsa-miR-34, hsa-miR-202, hsa-miR-205 and hsa-miR-30b. Secondly, hsa-miR-125b could help reduce the number of metastases in patients with NSCLC along with hsa-miR-133b, which correlates with tumour stage, degree of invasion, and EGFR expression, which may play an important role not only in prognostic but also in the treatment with drugs directed towards the *EGFR*. Finally, it has been described that the hsa-miR-128 correlates with high survival after treatment with gefitinib [18].

### miRNA as therapeutic biomarker

Platinum-derived treatments are the mainstay of chemotherapy treatment in patients with LC. However, sometimes we can observe a lack of response to the same. Different studies show us how some miRNAs can help predict the response to treatment, optimizing it to achieve personalized medicine. For example, hsa-miR-451 and hsa-miR-146b sensitize cells to cisplatin, hsa-miR- 326 is related to chemoresistance in AD [11], and hsa-miR-135b and hsa-miR-31 are related to resistance to treatment in AD. In addition, it has been observed how miRNAs are related to cancer metabolism by acting on some metabolic pathways, either directly or indirectly, such as hsa-miR-210, which regulates mitochondrial metabolism increased in the advanced stages of the disease. On the other hand, there is an excellent advance in immunotherapy thanks to the discovery of a transmembrane protein, programmed death-ligand 1 (PD-L1), found on the surface of neoplastic cells; therefore, the discovery of treatments directed against this protein helps to improve the situation of patients. Moreover, some miRNAs can guide us in the treatment. For example, hsa-miR-200 is strongly related to a high expression of PDL-1; however, hsa-miR-197 is inversely related to the expression of PDL-1 [9].

## Conclusion

Lung cancer has a high prevalence and mortality. However, thanks to the different advances in personalized medicine, biomarkers have been found that can be useful for early diagnosis, as is the case of the miRNAs. Many of them can be beneficial not only for diagnosis but also for treatment, as well as to elucidate the prognosis of these cancers. It is worth noting the great importance of discovering biomarkers that can be detected in more accessible samples and allowing a faster diagnosis, as is the case with liquid biopsies. Finding a biomarker, such as miRNAs, that can be analyzed in this type of sample is a considerable advance in these neoplasias.

## Data Availability

Not applicable.
